# DeepPhylo: Phylogeny‐Aware Microbial Embeddings Enhanced Predictive Accuracy in Human Microbiome Data Analysis

**DOI:** 10.1002/advs.202404277

**Published:** 2024-10-15

**Authors:** Bin Wang, Yulong Shen, Jingyan Fang, Xiaoquan Su, Zhenjiang Zech Xu

**Affiliations:** ^1^ School of Mathematics and Computer Sciences Nanchang University Nanchang 330031 China; ^2^ School of Information Engineering Nanchang University Nanchang 330031 China; ^3^ College of Computer Science and Technology Qingdao University Qingdao 266071 China; ^4^ State Key Laboratory of Food Science and Technology Nanchang University Nanchang 330077 China

**Keywords:** beta‐diversity, deep learning, microbiome, phylogeny

## Abstract

Microbial data analysis poses significant challenges due to its high dimensionality, sparsity, and compositionality. Recent advances have shown that integrating abundance and phylogenetic information is an effective strategy for uncovering robust patterns and enhancing the predictive performance in microbiome studies. However, existing methods primarily focus on the hierarchical structure of phylogenetic trees, overlooking the evolutionary distances embedded within them. This study introduces DeepPhylo, a novel method that employs phylogeny‐aware amplicon embeddings to effectively integrate abundance and phylogenetic information. DeepPhylo improves both the unsupervised discriminatory power and supervised predictive accuracy of microbiome data analysis. Compared to the existing methods, DeepPhylo demonstrates superiority in informing biologically relevant insights across five real‐world microbiome use cases, including clustering of skin microbiomes, prediction of host chronological age and gender, diagnosis of inflammatory bowel disease (IBD) across 15 studies, and multilabel disease classification.

## Graphical abstract

This study introduces DeepPhylo, a novel method that integrates abundance and phylogenetic information using phylogeny‐aware amplicon embeddings, enhancing both unsupervised discriminatory power and supervised predictive accuracy in microbiome data analysis. DeepPhylo outperforms existing methods across five real‐world microbiome applications, including clustering skin microbiomes, predicting host age and gender, diagnosing inflammatory bowel disease, and multilabel disease classification.

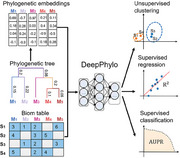

## 1 Introduction

The human microbiota, comprising bacteria, archaea, phages, and viruses, forms a complex ecosystem residing in various sites of the human body.^[^ ^1^, ^2^, ^3^ ^]^ Notably, changes in the microbiome have been found to correlate with the host's physiological status,^[^ ^3^, ^4^, ^5^, ^6^, ^7^, ^8^, ^9^, ^10^, ^11^, ^12^, ^13^, ^14^ ^]^ contributing to conditions like multiple sclerosis, type II diabetes, and IBD.^[^ ^15^, ^16^ ^]^ These changes are also influenced by factors such as age,^[^ ^17^ ^]^ living environment,^[^ ^18^ ^]^ and diet.^[^ ^3^, ^19^ ^]^

Advancements in 16S rRNA gene‐targeted sequencing have revolutionized microbiology by facilitating the cost‐effective identification of bacteria and archaea.^[^ ^20^, ^21^ ^]^ This technique focuses on sequencing the variable regions (V1‐V9) of the ribosomal RNA gene, which are subsequently processed into (OTUs) or Amplicon Sequence Variants (ASVs). The resulting microbial abundance table, along with a phylogenetic tree that delineates relationships between taxa, provides a rich dataset for downstream analysis.

Given the intimate association between the microbiome and human physiology, predictive modeling using microbiome data has emerged as a crucial area for exploring host phenotype and disease diagnosis.^[^ ^22^, ^23^, ^24^ ^]^ These predictive methods are typically categorized as unsupervised or supervised learning algorithms, based on their use of sample labels. Additionally, they can be distinguished as either phylogeny‐aware or phylogeny‐agnostic methods, depending on whether they integrate phylogenetic information.^[^ ^25^ ^]^ Machine learning methods in this context face challenges such as high dimensionality and sparsity of the data. Techniques like Lasso^[^ ^26^ ^]^ and random forest (RF),^[^ ^27^ ^]^ which are particularly effective for handling sparse data, are commonly employed.^[^ ^25^, ^28^, ^29^ ^]^ Recently, deep learning^[^ ^30^ ^]^ has shown significant potential in capturing complex patterns within microbiome data.^[^ ^31^, ^32^, ^33^ ^]^ Recent studies have sought to integrate both abundance and phylogenetic information for more insightful microbiome data analysis. For instance, PhyloRPCA^[^ ^34^ ^]^ expanded sample abundance data from leaf nodes to internal nodes of the phylogenetic tree for unsupervised data analysis. MDeep^[^ ^32^ ^]^ arranged abundance data according to phylogenetic relationships and employed 1D convolutional neural networks (CNNs) to capture the phylogenetic information between different OTUs. By transforming 1D abundance data into a 2D matrix, PopPhy‐CNN^[^ ^33^ ^]^ utilized 2D‐CNNs to capture the phylogenetic information across various branches of the phylogenetic tree. Additionally, Ph‐CNN^[^ ^35^ ^]^ enhanced traditional 1D‐CNNs by enabling the selection of the k‐nearest OTUs during the convolution process. However, these methods solely focus on the hierarchical structure of the phylogenetic trees while overlooking the quantitative distance information within them, thus limiting their capability to comprehensively capture the full breadth of phylogeny.

In this study, we introduce DeepPhylo, a novel machine learning approach designed to enhance sample clustering in unsupervised learning and improve predictive accuracy in supervised deep‐learning models by incorporating both phylogenetic and abundance information. DeepPhylo achieves this by extracting embeddings for each OTU and integrating them along with corresponding abundance data. Furthermore, by incorporating OTU embeddings into the neural network, DeepPhylo significantly improves the predictive performance of supervised deep learning models across various tasks, including regression, binary classification, and multilabel classification. The contributions of DeepPhylo are twofold: 1) it enhances unsupervised learning by incorporating phylogenetic embeddings derived from principal component analysis (PCA)^[^ ^36^ ^]^ techniques, thereby improving sample discriminatory power; and 2) it introduces a novel neural network architecture that effectively utilizes OTU embeddings to improve the predictive performance of host physiology using microbiome data.

## 2 Experimental Section

### 2.1 Encoding Phylogenetic Information by Phylogeny‐Aware OTU Embedding

The method integrated phylogenetic information into predictions through phylogeny‐aware embedding of OTUs. For a set of *m* OTUs, a phylogenetic tree was constructed to capture their phylogenetic relationships, yielding a phylogenetic distance matrix $D\in R^{m\times m}$. This matrix contained the pairwise patristic distances among all OTUs. PCA was applied to encode this phylogenetic information into embeddings as follows:

(1) $$X^{P}=\left[x_{1}^{P},x_{2}^{P},\text{…}x_{m}^{P}\right]=\mathrm{PCA}\left(D\right)$$

The patristic distance between two taxa was defined as the sum of the distances of their shortest connecting path on the phylogenetic tree.^[^ ^35^, ^37^ ^]^ In the experiments, the patristic distance was computed using the distance function from the BioPython package. For dimensionality reduction, the PCA implementation was employed from the scikit − learn package.

### 2.2 Phylogeny‐Aware OTU Embedding for Unsupervised Learning

**Figure 1:**
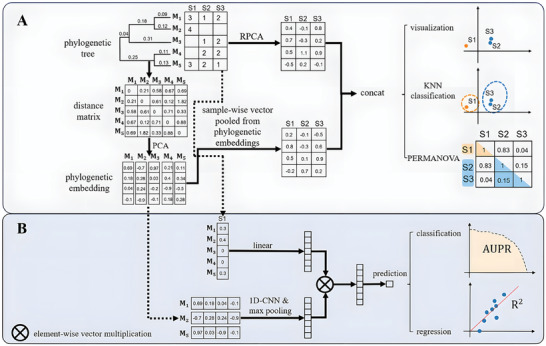
The overall framework of DeepPhylo. A) Enhancement of unsupervised learning with β‐diversity measures using phylogenetic information: Phylogenetic embeddings for each OTU are derived through PCA‐based dimensionality reduction of the phylogenetic distance matrix. These embeddings are then aggregated via summation pooling to encapsulate the phylogenetic relationships within the samples. Simultaneously, the sample abundance matrix undergoes dimensionality reduction using RPCA to extract abundance‐related features. The resulting features from both sets are then concatenated to create a fused feature embedding that integrates phylogenetic and abundance information. B) Supervised deep learning model integrating both abundance and phylogenetic information: the model is structured with two primary input modules: a linear input module that processes sample abundance data, and a convolutional input module that processes phylogenetic OTU embeddings. The outputs from these modules are combined to form a comprehensive feature representation, which is then used for the downstream predictive modeling tasks.

To enhance unsupervised learning performance, phylogenetic information alongside taxon abundance information was incorporated to achieve a comprehensive representation of samples (see **Figure** **1**A). Formally, for a study involving *n* samples and *m* phylogeny‐aware OTU embeddings, an abundance matrix $X^{A}=\left[x_{1}^{A},x_{2}^{A},\text{…},x_{n}^{A}\right]\in R^{m\times n}$ and a phylogenetic matrix $X^{P}=\left[x_{1}^{P},\;x_{2}^{P},\text{…},x_{m}^{P}\right]\in R^{m\times d}$ were used. The abundance matrix **X** ^A^ was high‐dimensional and sparse, while the phylogenetic matrix **X** ^P^ was low‐dimensional and dense. RPCA^[^ ^38^ ^]^ was applied to reduce the dimensionality of the abundance matrix **X** ^A^ to obtain low‐dimensional dense features $\left[f_{1}^{A},f_{2}^{A},\text{…},f_{n}^{A}\right]$, representing abundance information for each sample. Simultaneously, sample‐wise summation pooling across the presented OTUs within each sample was utilized to derive phylogeny‐related feature $\left[f_{1}^{P},f_{2}^{P},\text{…},f_{n}^{P}\right]$ for each sample. This pooling method involved summing the phylogenetic embeddings for each OTU present in a sample to obtain a single vector representing the phylogenetic profile of that sample. Finally, the study concatenated the abundance and phylogeny features to create a fused representation of each sample. These fused features captured both the abundance and phylogenetic information, which was expected to improve the performance of microbial analyses.

### 2.3 Phylogeny‐Aware OTU Embedding for Deep Learning Model

#### 2.3.1 Overview of DeepPhylo Deep Learning Model

DeepPhylo deep learning model was composed of three modules: an input module, a feature fusion module, and an output module. As illustrated in Figure 1B, DeepPhylo employed a dual‐tower architecture that processes abundance features and phylogenetic features simultaneously. This structure enabled the model to effectively integrate these two types of features for predictive tasks.

#### 2.3.2 Input Module

DeepPhylo incorporated two distinct input modules: a linear input module for handling abundance features ***x*** ^A^, and a convolutional input module for processing phylogenetic information **X** ^P^. The linear input module encoded high‐dimensional and sparse abundance features into low‐dimensional and dense vectors ***f*** ^A^, according to the transformation Equation (2). The convolutional module was designed to leverage phylogeny‐aware embeddings. It organized the embeddings of observed OTUs within each sample into a matrix, $X^{P}=\left[x_{1}^{P},\;x_{2}^{P}\text{…}\right]$. Through the application of multiple 1D convolutional filters, the model captured phylogenetic correlations among taxa, identifying important OTU patterns. Subsequently, a MaxPooling layer condensed feature embedding ***f*** ^P^, extracting the most salient feature from each filter.

(2) $$f^{A}=a\left(Wx^{A}+b\right)$$

(3) $$f^{P}=\mathrm{MaxPool}\left(F^{P}\right)$$

(4) $$F_{ij}^{P}=a\left(\sum_{k=1}^{l}W_{kj}x_{i+k-1}^{P}+b_{j}\right)$$

where $F_{ij}^{P}$ is the entry of the phylogenetic feature map **F** ^P^ corresponding to the *i*‐th taxon present in ***x*** ^A^ and the *j*‐th filter, *l* is the filter size, **W**_*kj*_ are the parameters of the *j*‐th convolution filter, and *a* is the activation function.

#### 2.3.3 Feature Fusion Module and Output Module

The feature fusion module of DeepPhylo integrated the abundance features ***f*** ^A^, and phylogenetic features ***f*** ^P^, using element‐wise multiplication to produce the fused feature embedding ***f***. In the output layer, this fused feature embedding was processed by one or more linear layers to predict a single output, *s*. For regression tasks, *s* served as the final output, while for binary classification tasks, *s* was further passed through a sigmoid activation function to yield the prediction probability *p*(*y*  =  1| ***x***).

(5) $$f=f^{A}⊗f^{P}$$

(6) s=Wf+b

where ⊗ is element‐wise multiplication of two vectors.

For regression tasks, the Mean Squared Error (MSE) loss function *l* is computed as $l=\frac{1}{n}\;\sum_{i=1}^{n}{\left(y_{i}-{\hat{y}}_{i}\right)}^{2}$, where *y_i_* is the true label and ${\hat{y}}_{i}$ is the predicted label, and *n* is the number of samples. For binary classification tasks, the Binary Cross‐Entropy (BCE) loss function *l* is calculated as $l=-\frac{1}{n}\sum_{i=1}^{n}\left(y_{i}\log p\left(\;y_{i}=1|x\right)+\left(1-y_{i}\right)\log p\left(\;y_{i}=0|x\right)\right)$. During training, the AdamW^[^ ^39^ ^]^ optimizer was employed to update network parameters, with a weight decay hyperparameter λ for L2 regularization. Thus, the objective function was $\min_{W}\left\{l+\lambda ∥W{∥}^{2}\right\}$ and the gradients were obtained through back‐propagation during training phase.

### 2.4 Performance Evaluation Metrics

For regression, R^2^ was employed as the assessment criterion. For binary classification, sensitivity, specificity, accuracy, precision, Matthews Correlation Coefficient (MCC), and F1 score were used. The definitions of these metrics are as follows:

(7) $$\mathrm{Sensitivity}=\frac{\mathrm{TP}}{\mathrm{TP}+\mathrm{FN}}$$

(8) $$\mathrm{Specificity}=\frac{\mathrm{TN}}{\mathrm{TN}+\mathrm{FP}}$$

(9) $$\mathrm{Accuracy}=\frac{\mathrm{TP}+\mathrm{TN}}{\mathrm{TP}+\mathrm{TN}+\mathrm{FP}+\mathrm{FN}}$$

(10) $$\mathrm{Precision}=\frac{\mathrm{TP}}{\mathrm{TP}+\mathrm{FP}}$$

(11) $$F1=\frac{2\times \mathrm{Precision}\times \mathrm{Recall}}{\mathrm{Precision}+\mathrm{Recall}}$$

(12) $$\mathrm{MCC}=\frac{\mathrm{TP}\times \mathrm{TN}-\mathrm{FP}\times \mathrm{FN}}{\sqrt{\left(\mathrm{TP}+\mathrm{FN}\right)\times \left(\mathrm{TP}+\mathrm{FP}\right)\times \left(\mathrm{TN}+\mathrm{FN}\right)\times \left(\mathrm{TN}+\mathrm{FP}\right)}}$$

where TP and TN represent the counts of accurately classified positive and negative samples, respectively, while FN and FP denote the counts of misclassified positive and negative samples in the confusion matrix. Note that Recall is equivalent to Sensitivity in this context.

### 2.5 Datasets and Benchmark

The deep learning models, DeepPhylo and NN, were implemented using the PyTorch package in Python. Other deep learning based comparison methods, including MDeep, PopPhy‐CNN, and Ph‐CNN, were run using their provided source code. RF and Lasso algorithms were run in Python using the scikit‐learn package. Hyperparameter tuning for all methods was conducted as detailed in Table S1 (Supporting Information).

In this study, the effectiveness of the proposed DeepPhylo method is evaluated using five real‐world datasets, encompassing both unsupervised and supervised use cases. For the unsupervised analysis, the approach described in ref.[34] was followed and human skin microbiome samples collected from villages in South America were utilized. For the supervised tasks, the study tested DeepPhylo on the datasets including human age regression and gender classification of human twins from,^[^ ^32^ ^]^ IBD diagnosis from,^[^ ^31^ ^]^ and a multilabel disease classification dataset from Guangdong Gut Microbiome Project (GGMP).^[^ ^40^, ^41^ ^]^

To ensure rigorous evaluation, the datasets for the human age regression, twins gender classification, and multilabel disease classification tasks were split into training, validation, and test sets in a ratio of 68:12:20, allowing independent test set evaluation. For the IBD dataset, which included multiple studies, a Leave‐One‐Dataset‐Out (LODO) evaluation^[^ ^31^ ^]^ was employed to enhance robustness. In this approach, each study's samples were used as a test set in turn, while the remaining data served as the training set.

The data preprocessing for all five datasets followed the procedures specified in the respective source publications. A comprehensive summary of the datasets, including sample sizes, accession numbers, and other relevant details, is provided in Table S2 (Supporting Information).

### 2.6 Simulation Studies

#### 2.6.1 Simulation Strategy

Comprehensive simulation studies were conducted to rigorously assess the predictive performance of DeepPhylo in binary classification tasks, benchmarking it against other established methods. The simulation approach was carefully designed to align with methodologies used in previous studies.^[^ ^32^, ^42^ ^]^ Specifically, a total of 400 synthetic microbial samples were generated using the NorTA (Normal To Anything) approach,^[^ ^43^, ^44^ ^]^ which effectively captures the complexity and inherent correlations of real microbial community data. The statistical parameters used for these simulations were derived from an authentic microbial dataset,^[^ ^45^ ^]^ ensuring that the synthetic data accurately mirrored real‐world conditions. The 400 generated samples, each containing 927 OTUs, were subsequently divided into training, validation, and test sets in a 200:100:100 ratio. OTU counts were normalized by total sample counts to yield relative abundances. Binary outcomes were simulated based on the abundance profiles of outcome‐associated OTUs (aOTUs), with parameters cluster size, signal density, and phylogenetic informativeness of aOTUs, following the methodology outlined in.^[^ ^32^, ^42^ ^]^ For a detailed introduction to these parameters, please refer to the original studies.^[^ ^32^, ^42^ ^]^ The study systematically investigated how variations in the phylogenetic tree influenced the prediction performance, as conducted in the previous studies.^[^ ^32^, ^42^ ^]^

The simulation parameters were defined as follows:

- Cluster size: aOTUs were clustered at varying phylogenetic depths, leading to outcome‐associated clusters (aClusters) of different sizes. OTUs were grouped into 50, 20, and 10 clusters to represent small, medium, and large aClusters, respectively.
- Signal Density: The proportion of aClusters relative to the total number of clusters was varied at 10%, 20%, and 40%, corresponding to low, medium, and high signal densities, respectively.
- Phylogenetic informativeness: For informative trees, aOTUs within each aCluster were assigned consistent directional effects on the outcome. Conversely, in non‐informative trees, adjacent aOTUs within an aCluster were assigned opposite effects, thereby violating the assumption that phylogenetically related aOTUs share similar functional roles and effects.

The binary outcome 𝑦𝑖 for each sample 𝑖 was generated from a Bernoulli distribution with a probability 𝑝𝑖, which was calculated using the sigmoid function applied to the aggregated linear effects 𝜂𝑖.

(13) $$y_{i}∼\mathrm{Bern}\left(p_{i}\right)$$

(14) $$p_{i}=Sigmoid\;\left(\eta_{i}\right)=\frac{1}{1+e^{-\eta_{i}}}$$

(15) $$\begin{array}{c} \eta_{i}=\left\{\begin{array}{cc} \sum_{k=1}^{K}\sum_{j\in C_{k}}\beta_{k}x_{ij}, & if\;informative\\ \sum_{k=1}^{K}\left(\sum_{j\in C_{k}^{inv}}-\beta_{k}x_{ij}+\sum_{j\in C_{k}\setminus C_{k}^{inv}}\beta_{k}x_{ij}\right) & else \end{array}\right. \end{array}$$

(16) $$C_{k}^{inv}∼\mathrm{RandomSample}\left(C_{k},\;\frac{\left|C_{k}\right|}{2}\right)$$

(17) $$\beta_{k}∼N\left(0,\;{\sigma_{\beta }}^{2}\right)$$

where the effect factor β_*k*_ was sampled from a centered normal distribution, allowing the effect of an aCluster to be either positive or negative, with σ_β_^2^ set to 4. $C_{k}$ was the set containing indices of the *k*th aCluster among total *K* aClusters (k∈{1,2,3…,K}), with *x_ij_* representing the *j*th OTU abundance in sample *i*. For non‐informative trees, the effect factor β_*k*_ for half of the aOTUs, denoted as $C_{k}^{inv}$, in each aCluster was inverted.

## 3 Results

### 3.1 Integrating Microbial Abundance Data and Phylogeny‐Aware Embedding by DeepPhylo

As shown in Figure 1, DeepPhylo significantly enhances the discriminative power of microbial data in unsupervised analyses and improves predictive accuracy in supervised learning tasks by integrating phylogeny‐aware amplicon embeddings with abundance information. This integration addresses the inherent complexity of microbiome data, which is characterized by high dimensionality and sparse distributions.

In the unsupervised context, DeepPhylo begins by constructing phylogeny‐aware amplicon embeddings from the phylogenetic tree. These embeddings capture the evolutionary relationships among OTUs, providing a compact representation of the phylogenetic structure. For each sample, these embeddings are then aggregated to encapsulate the overall phylogenetic profile of the microbial community within that sample. Meanwhile, the microbial abundance data undergo dimensionality reduction via Robust Aitchison PCA (RPCA),^[^ ^38^ ^]^ a method known for its effectiveness in handling compositional data, yielding a low‐dimensional feature representation that captures the abundance‐related variations across samples. The phylogenetic and abundance‐derived embeddings are subsequently concatenated to create a fused feature vector, which enhances the model's ability to differentiate between samples.

In the supervised deep learning context, DeepPhylo employs a dual‐tower architecture to process two distinct types of feature vectors through complementary pathways. The abundance features are fed into a linear input module, while the phylogenetic features are processed through a convolutional input module. The convolutional module applies 1D convolutional filters to capture local patterns and correlations among related OTUs. This convolutional operation was performed on the phylogeny‐aware embeddings along OTU direction, with the relative positions of OTU embedding aligned according to their positions in the phylogenetic tree. By modeling complex interactions between phylogeny and abundance, DeepPhylo ultimately enhances its predictive performance.

#### 3.1.1 DeepPhylo Demonstrates Robust Performance on Simulated Data

To evaluate the impact of phylogenetic signals on DeepPhylo's performance under various phylogenetic conditions, we conducted a series of simulation experiments. These studies assessed how changes in evolutionary cluster sizes, signal densities, and the informativeness of the phylogenetic tree influenced model accuracy.

DeepPhylo consistently demonstrated superior accuracy across all simulation scenarios, regardless of cluster size, signal density, or phylogenetic informativeness. This consistent performance underscores DeepPhylo's robustness in effectively leveraging phylogenetic information for microbial data analysis. MDeep and PopPhy‐CNN also showed strong stability, particularly under medium to high signal densities, where their performance was comparable to DeepPhylo. However, Ph‐CNN, despite showing good performance in some cases, lacked overall consistency. In contrast, RF, Lasso, and NN generally underperformed, especially in scenarios with medium to high phylogenetic signals, highlighting the critical role of incorporating phylogenetic information into machine learning models. These findings collectively highlight the effectiveness of DeepPhylo across a range of simulated conditions, confirming its potential as a powerful tool for microbial data analysis.

### 3.2 Phylogenetic Information Enhances Unsupervised Sample Clustering

#### 3.2.1 Case Study 1: Skin Microbiome Samples in South America

**Figure 2:**
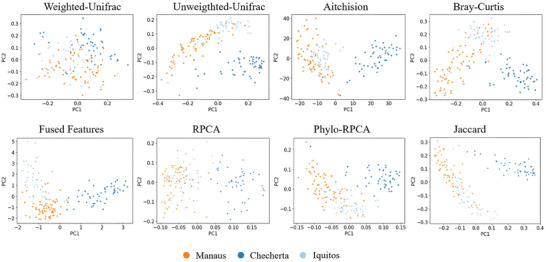
PCA scatter plot visualizations of samples using the benchmarked methods.

**Figure 3:**
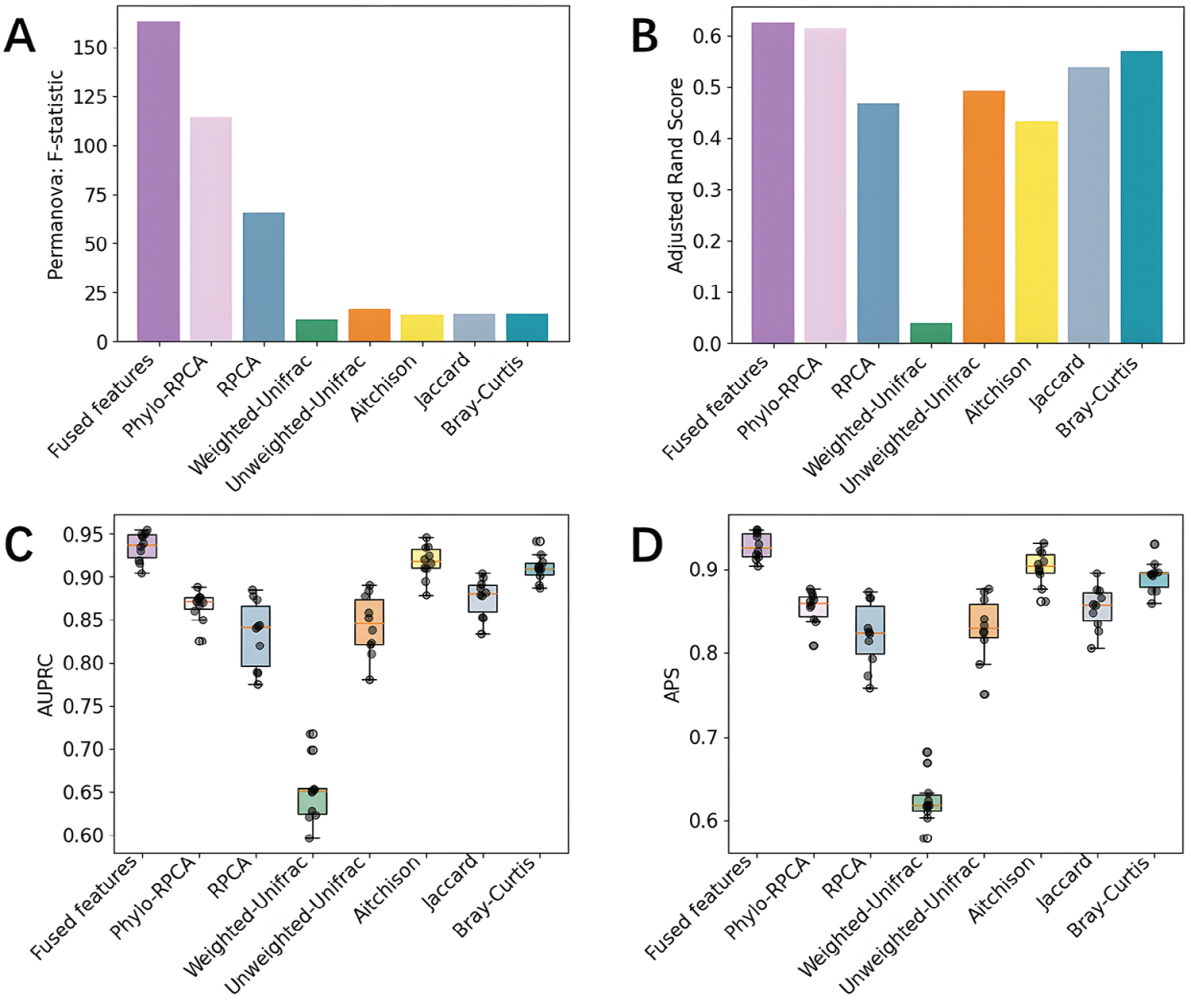
Quantitative evaluation of clustering performance: A) PERMANOVA F‐statistic. B) Adjusted Rand Index of K‐means clustering. C,D) Area Under the Precision‐Recall Curve (AUPRC) and Average Precision Score (APS) of tenfold KNN classification.

DeepPhylo's efficacy in unsupervised microbiome analysis was benchmarked using 16S data amplicon sequencing of 164 South American skin samples,^[^ ^18^ ^]^ following the exact approach described in^[^ ^34^ ^]^ for direct comparison and consistent evaluation. Compared to the PCoA analysis based on various commonly used beta‐diversity metrics, including RPCA,^[^ ^38^ ^]^ phylo‐RPCA,^[^ ^34^ ^]^ Jaccard,^[^ ^46^ ^]^ Weighted‐Unifrac,^[^ ^47^ ^]^ Unweighted‐Unifrac,^[^ ^48^ ^]^ Aitchison,^[^ ^49^ ^]^ and Bray‐Curtis,^[^ ^50^ ^]^ DeepPhylo outperformed them across all metrics. The scatter plots in **Figure** **2** illustrate that DeepPhylo's inclusion of phylogenetic information results in more distinct clustering by city than the other methods. To quantify these observations, permutational multivariate analysis of variance (PERMANOVA),^[^ ^51^ ^]^ K‐means clustering, and KNN classification were performed. As shown in **Figure** **3**, DeepPhylo demonstrates significant improvement in the F‐statistic metric compared to the phylogeny‐aware Phylo‐RPCA and other phylogeny‐agnostic methods. Specifically, the K‐means clustering performance of DeepPhylo is superior to that of the other methods evaluated (Figure 3B). The fused features of DeepPhylo excel in sample classification using the KNN algorithm (Figure 3C,D). In conclusion, the comprehensive evaluation consistently supports the superior performance of DeepPhylo in unsupervised clustering and classification tasks. Notably, Phylo‐RPCA demonstrates improvement over standard RPCA, corroborating the notion that integrating phylogenetic information aids in uncovering microbiome patterns.

### 3.3 Phylogenetic Information Enhances Predictive Deep Learning Models

In the supervised assessment, we compared DeepPhylo with MDeep, PopPhy‐CNN, Ph‐CNN, Feed‐forward Neural Network (NN), RF, and Lasso across four real‐world datasets, including host chronological age regression, gender classification, IBD diagnosis, and GGMP multilabel classification tasks. MDeep performed well in the age and gender datasets in a previous study,^[^ ^32^ ^]^ by leveraging the evolutionary information through a 1D‐CNN. PopPhy‐CNN used 2D CNN on metagenomic data by transforming metagenomic abundance data to a 2D matrix.^[^ ^33^ ^]^ Ph‐CNN modified convolutional calculations to incorporate phylogenetic information.^[^ ^35^ ^]^ NN is a simple feedforward neural network designed with the same number of fully connected layers as DeepPhylo. RF and Lasso are two well‐established machine learning algorithms for microbiome data with unique advantages: RF ensembles multiple decision trees for robust prediction and Lasso is particularly effective in managing feature sparsity.^[^ ^25^, ^28^, ^29^ ^]^

#### 3.3.1 Case Study 2: Host Chronological Age Prediction with Gut Microbiome

**Figure 4:**
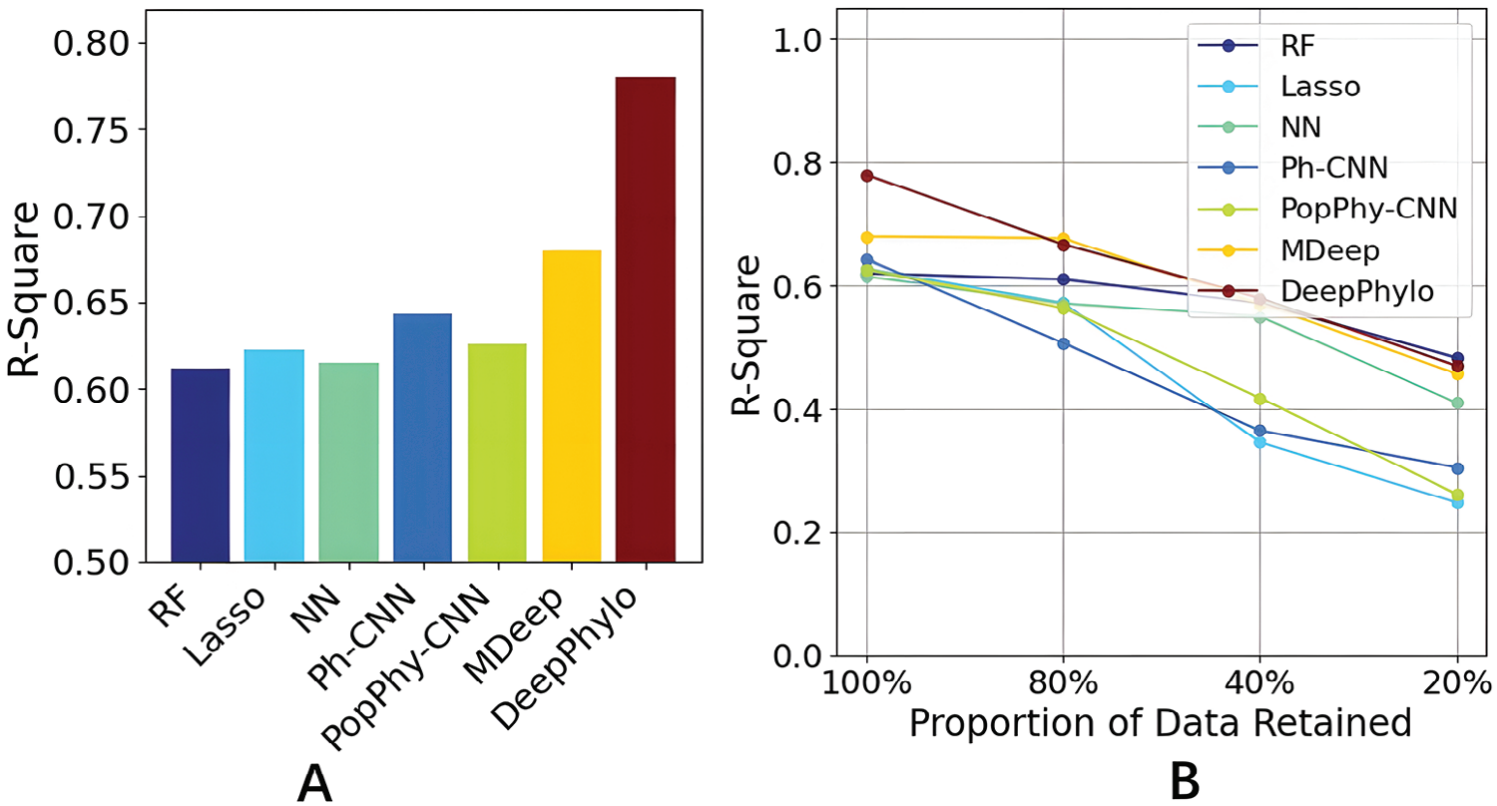
Prediction performance of chronological age using gut microbiome of individuals: A) *R* ^2^ of different methods. B) Effect of reducing the proportion of training data on model performance.

We analyzed a dataset^[^ ^32^ ^]^ of 308 individuals, each profiled with 1087 OTUs, for chronological age prediction. The *R* ^2^ values were computed to evaluate the prediction accuracies of each method. As shown in **Figure** **4**A, DeepPhylo outperforms the other methods with the best *R* ^2^. The methods incorporating phylogenetic information, including DeepPhylo, MDeep, PopPhy‐CNN, and Ph‐CNN, outperformed those that did not—such as NN, RF, and Lasso, highlighting the advantage of integrating phylogenetic information to improve age prediction accuracy.

Machine learning, particularly deep learning, typically requires large datasets for effective training models. In this study, we further explored the robustness of these models with reduced training data sizes to assess their performance. As illustrated in Figure 4B, all methods exhibited varying degrees of performance degradation with decreasing training data sizes. When the training sample size was reduced to 20% of the original dataset, resulting in 41 training samples, the predictive performance of Lasso, Ph‐CNN, and PopPhy‐CNN declined significantly. The pronounced performance decline in Lasso could be attributed to its sparse feature selection mechanism, which may lead to an overly simplified model when applied to small datasets. The reduced performance of other deep learning models, including NN, Ph‐CNN, and PopPhy‐CNN, with small samples, may suggest an increased risk of overfitting under such conditions. In contrast, DeepPhylo, MDeep, and RF were more robust with significantly reduced training sample sizes.

#### 3.3.2 Case Study 3: Host Gender Prediction Using Gut Microbiome from Human Twins

Previous research has established a link between the composition of the human gut microbiota and the host's gender. For the gender prediction task, we utilized a dataset from the study,^[^ ^32^ ^]^ comprising 995 individuals profiled with 16S rRNA amplicon sequencing. We evaluated the classification performance using the following metrics: Accuracy, MCC, F1 score, Precision, Sensitivity, and Specificity.

**Figure 5:**
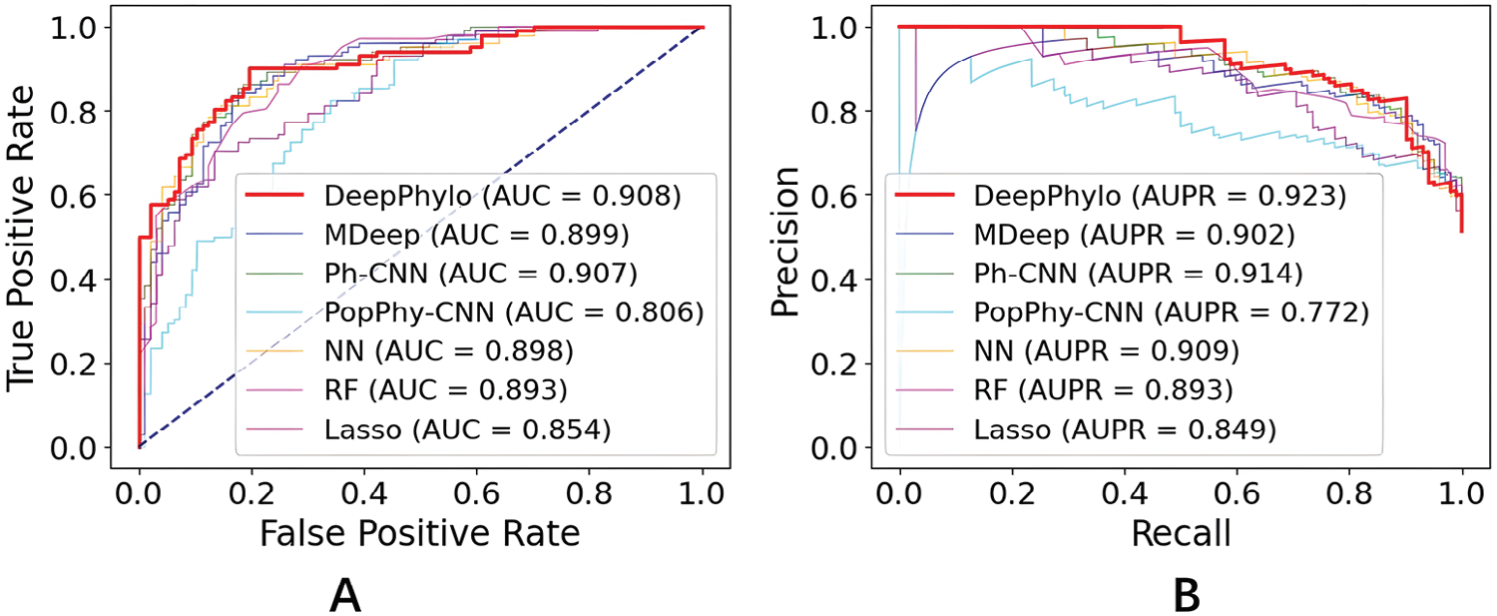
Receiver Operating Characteristic (ROC) curves and Precision‐Recall curves for binary prediction of host gender.

As shown in **Table** **1**, our results revealed that deep learning approaches, including DeepPhylo, MDeep, Ph‐CNN, and NN, generally outperformed machine learning methods RF and Lasso, with the exception of PopPhy‐CNN, which achieved performance comparable to RF. Notably, DeepPhylo excelled across key evaluation metrics including Accuracy, MCC, and F1‐score. While MDeep and Ph‐CNN exhibited a slight advantage in Sensitivity, Specificity, and Precision metrics, likely reflecting a trade‐off where various methods prioritize certain metrics at the expense of others. The superiority of DeepPhylo is also shown by the Precision‐Recall and Specificity‐Sensitivity curves (**Figure** **5**A,B), where DeepPhylo achieved the highest AUC and AUPR values.

**Table 1:** Prediction performance in binary prediction of host gender. The method with the highest performance is highlighted in bold, while the second‐best performing method is underlined.

	RF	Lasso	NN	Ph‐CNN	PopPhy‐CNN	MDeep	DeepPhylo
Accuracy	0.792	0.770	0.814	0.839	0.789	0.829	0.849
MCC	0.586	0.538	0.627	0.678	0.581	0.665	0.701
F1 score	0.809	0.783	0.821	0.845	0.806	0.846	0.856
Precision	0.774	0.767	0.795	0.836	0.763	0.788	0.814
Specificity	0.732	0.735	0.763	0.824	0.722	0.742	0.804
Sensitivity	0.850	0.802	0.873	0.852	0.853	0.911	0.902

#### 3.3.3 Case Study 4: Gut Microbiome‐Based Diagnosis of IBD

The human gut microbiome is associated not only with physiological characteristics such as age and gender but also with various diseases. To assess the effectiveness of DeepPhylo in diagnosing IBD, a data set from,^[^ ^31^ ^]^ consisting of 7056 samples across 15 diverse studies, was collected for LODO evaluation. In this LODO scheme, each dataset was sequentially excluded from the training set and used exclusively for testing.

**Figure 6:**
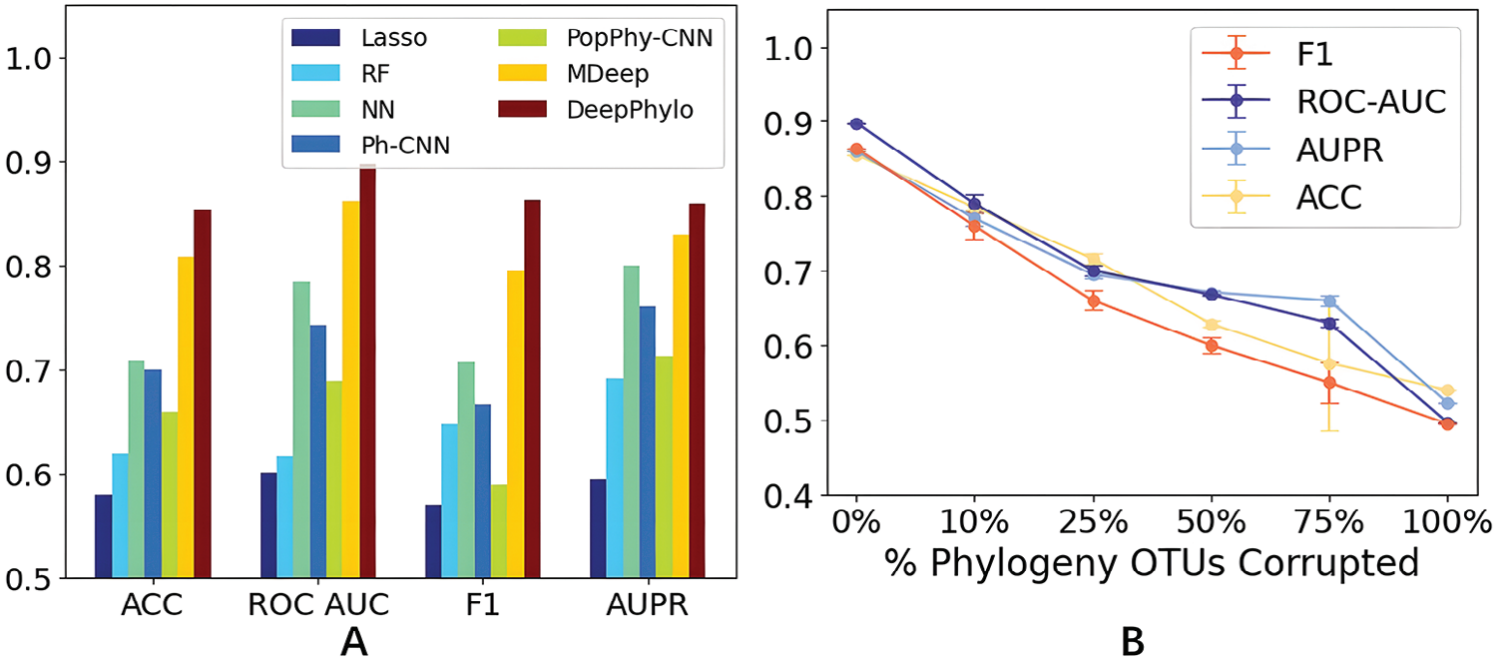
Evaluation of prediction performance for the gut microbiome‐based diagnosis of IBD. A) Comparison of performance metrics (ACC, ROC‐AUC, AUPR, F1) across various methods. B) Analysis of performance impact when varying proportions (10%, 25%, 50%, 75%, and 100%) of phylogenetic signals are removed. The analysis was repeated ten times to account for the randomness in selecting OTUs for the removal of phylogenetic signal at each proportion.

As shown in **Figure** **6**A, DeepPhylo achieved superior performance across all classification metrics, demonstrating its robustness in IBD diagnosis. Notably, DeepPhylo and MDeep consistently achieved higher accuracy compared to traditional methods such as RF, Lasso, and NN. In contrast, PopPhy‐CNN and Ph‐CNN underperformed relative to NN. These findings suggest that both DeepPhylo and MDeep are particularly effective in leveraging phylogenetic signals to improve diagnostic accuracy.

To further investigate the role of phylogenetic information in the performance of the DeepPhylo model, we conducted an additional experiment. We systematically removed the phylogenetic signal from varying percentages (10%, 25%, 50%, 75%, and 100%) of OTU embeddings. As shown in Figure 6B, there was a consistent decline in performance as the phylogenetic signal was diminished. This decline highlights the importance of accurately incorporating phylogenetic relationships into the model, confirming that DeepPhylo significantly benefits from these relationships in achieving superior classification performance.

#### 3.3.4 Case Study 5: Gut Microbiome‐Based Multilabel Disease Classification

To further assess the diagnostic capability of DeepPhylo in complex and realistic scenarios, we extended its application to a more challenging task: the simultaneous classification of multiple diseases using human gut microbiome data. For this analysis, we utilized data from the Guangdong Gut Microbiome Project (GGMP),^[^ ^40^, ^41^ ^]^ which includes 5347 subjects. Within this cohort, 1293 individuals were diagnosed with metabolic syndrome (MetS), 935 with gastritis, 551 with T2DM, and 190 with gout. The dataset allowed for each individual to be labeled with multiple conditions simultaneously, enabling a thorough assessment of DeepPhylo's performance in a multilabel classification context. Due to the imbalanced nature of this dataset, we selected AUC and AUPR as the primary metrics to assess the predictive performance of different methods across all diseases as well as for each individual condition.

As shown in **Table** **2**, DeepPhylo consistently outperformed the other models across most metrics, with the exception of the AUC‐Gastritis and AUPR‐Gout, where its performance was comparable to PopPhy‐CNN and MDeep, respectively. Compared to RF, Lasso, and NN, the models incorporating phylogenetic information—DeepPhylo, MDeep, PopPhy‐CNN, and Ph‐CNN—demonstrated superior performance, indicating the effectiveness of integrating phylogenetic information in predicting multiple diseases. Furthermore, NN outperformed RF and Lasso, suggesting that more complex neural network architectures are better suited for capturing critical features in multilabel disease classification. Despite DeepPhylo outperforming comparative methods, its moderate predictive accuracy suggests that further research is necessary. This limitation may stem from shared microbiome changes across various diseases or a weak association between certain diseases and the microbiome, particularly in the context of complex multilabel classification and imbalanced training data. These results highlight DeepPhylo's capability in handling complex multilabel classification tasks in microbiome research, showcasing its ability to effectively leverage phylogenetic information for superior predictive performance.

**Table 2:** Prediction performance in multilabel disease classification. The method with the highest performance is highlighted in bold, while the second‐best performing method is underlined.

	RF	Lasso	NN	Ph‐CNN	PopPhy‐CNN	MDeep	DeepPhylo
AUC‐All	0.504	0.629	0.660	0.691	0.7	0.696	0.705
AUC‐MetS	0.575	0.538	0.571	0.58	0.583	0.566	0.599
AUC‐Gastritis	0.521	0.513	0.522	0.517	0.547	0.53	0.545
AUC‐T2DM	0.549	0.563	0.547	0.594	0.57	0.59	0.606
AUC‐Gout	0.584	0.556	0.533	0.548	0.66	0.662	0.663
AUPR‐All	0.151	0.214	0.225	0.259	0.256	0.261	0.272
AUPR‐MetS	0.320	0.29	0.311	0.328	0.309	0.333	0.339
AUPR‐Gastritis	0.186	0.194	0.189	0.19	0.2	0.191	0.213
AUPR‐T2DM	0.138	0.122	0.116	0.14	0.125	0.132	0.141
AUPR‐Gout	0.068	0.059	0.05	0.045	0.071	0.074	0.072

## 4 Discussion

In this study, we introduced DeepPhylo, a novel approach designed to enhance the predictive capabilities of microbial data by incorporating phylogenetic information into microbial abundance profiles. DeepPhylo leverages PCA to effectively encode the phylogenetic relationships among OTUs as OTU embeddings. During unsupervised learning, these embeddings are aggregated within each sample and combined with its abundance features, resulting in fused features that significantly enhance discriminative power between different sample groups. In the supervised learning scenario, a 1D‐CNN is employed to capture crucial OTU patterns within the phylogeny, which are then integrated with abundance features to improve the model's predictive accuracy. The effectiveness of DeepPhylo was validated through experiments conducted on five real‐world datasets.

The importance of incorporating phylogenetic information into microbial data analysis has long been recognized, as evidenced by methods such as UniFrac,^[^ ^47^, ^48^ ^]^ which leverage evolutionary relationships to assess microbial community differences. Extending this concept to machine learning and deep learning models is a logical progression, as it enables the capture of complex relationships that may be overlooked when relying solely on abundance data. By integrating phylogenetic information within these advanced models, we can gain deeper insights into the underlying biology and enhance the predictive performance of these computational approaches. However, integrating phylogeny with abundance information poses a considerable challenge due to their inherent heterogeneity. While neural networks typically perform well with large datasets, the batch effects in microbial data may disrupt the expected positive correlation between dataset size and model performance.^[^ ^25^, ^26^ ^]^ This observation implies that simply combining diverse data from multiple studies may reduce predictive accuracy. Therefore, it is essential to develop a robust foundational model that can benefit from the diversity of large datasets, thereby extending the predictive capabilities of deep learning in the analysis of microbial data.

## Conflict of Interest

The authors declare no conflict of interest.

## Author Contributions

Z.Z.X. conceived the study and supervised the project. B.W. developed software and conducted analyses. Y.S. curated datasets and assessed the benchmarks. X.S. contributed to dataset curation. All authors participated in the discussion of the results and their implications. B.W. and Z.Z.X collaboratively wrote the manuscript.

## Supporting information



Supporting Information

## Data Availability

The data that support the findings of this study are available from the corresponding author upon reasonable request.
