# Supplementary material for: DeepPhylo: Phylogeny‐Aware Microbial Embeddings Enhanced Predictive Accuracy in Human Microbiome Data Analysis
Source: Adv Sci (Weinh). 2024 Oct 15;11(45):2404277. doi: 10.1002/advs.202404277 (PMC11615782; doi:10.1002/advs.202404277)
Supplement: Supplementary file 1 — Supporting Information [file ADVS-11-2404277-s001.docx]

# Supplementary Materials

Supplementary Table S1 The hyperparameter combinations for model tuning in supervised tasks.

|  | machine learning hyperparameters | deep learning hyperparameters | | |
| --- | --- | --- | --- | --- |
|  | - | Optimizer | NN hyperparameters | Convolution hyperparameters |
| Lasso | Alpha $\in${1e-8, 1e-7,…1e-3} | - | - | - |
| RF | n_tree $\in${200} | - | - | - |
| NN | - | learning_rate$\in${1e-3, 1e-4}  batch_size$\in${32,64} | hidden_size$\in${16,32,64}  activation$\in${ ReLU,Tanh,Sigmoid}  dropout$\in${0.0,0.2,0.5} | - |
| Ph-CNN | - | learning_rate$\in${1e-3, 1e-4}  batch_size$\in${32,64} | hidden_size$\in${64, 128}  dropout$\in${0.0,0.2,0.5} | nb_filters$\in${[16,16]**,** [12,12] }  phylo_neighbours $\in${[4,4], [8,8]} |
| PopPhy-CNN | - | learning_rate$\in${1e-3,1e-4}  batch_size$\in${32,64} | hidden_size$\in${16,32,64}  dropout$\in${0.0,0.2,0.5} | nb_filters$\in${32**,** 64 }  kernel_shape$\in${[5,9]**,** [5,7], [3,5]} |
| MDeep | - | learning_rate$\in${1e-4}  batch_size$\in${32,64} | hidden_size$\in${16,32,64}  activation$\in${ ReLU,Tanh,Sigmoid}  dropout$\in${0.0,0.2,0.5} | nb_filters1$\in${64**,**128,256}  conv_kernel_size1$\in${32,64}  nb_filters2$\in${4,6}  conv_kernel_size2$\in${4,2} |
| DeepPhylo | - | learning_rate$\in${1e-4}  batch_size$\in${32,64} | hidden_size$\in${16,32,64}  activation$\in${ReLU,Tanh,Sigmoid}  dropout$\in${0.0,0.2,0.5} | nb_filters1$\in${16,32,64}  conv_kernel_size{7,9,11}  conv_poolsize$\in${2,4,6} |

Supplementary Table S2 Summary of datasets used in this study with corresponding literature and accession number.

| task name | literature | dataset source | number of samples | number of OTUs |
| --- | --- | --- | --- | --- |
| Unsupervised clustering | [34] | QIITA10333 | 164 | 1443 |
| Age regression | [32] | QIITA850 | 308 | 1087 |
| Twins gender binary classification | [32] | QIITA737 | 955 | 2291 |
| IBD diagnosis | [31] | PRJEB11419 | 1494 | 1218 |
|  |  | PRJNA308319 | 39 | 874 |
|  |  | PRJEB13680 | 259 | 829 |
|  |  | PRJEB13679 | 1192 | 957 |
|  |  | PRJEB23009 | 217 | 759 |
|  |  | ibdmdb.org | 147 | 703 |
|  |  | PRJNA317429 | 83 | 209 |
|  |  | PRJNA418765 | 579 | 751 |
|  |  | PRJNA436359 | 1142 | 909 |
|  |  | PRJEB13895 | 962 | 997 |
|  |  | PRJEB13619 | 58 | 853 |
|  |  | PRJEB14674 | 332 | 1038 |
|  |  | PRJEB13051 | 23 | 768 |
|  |  | PRJEB6518 | 513 | 564 |
|  |  | PRJEB19825 | 25 | 273 |
|  |  | Total | 7065 | 1522 |
| multilabel disease classification | [41] | PRJEB18535 | 5347 | 449 |


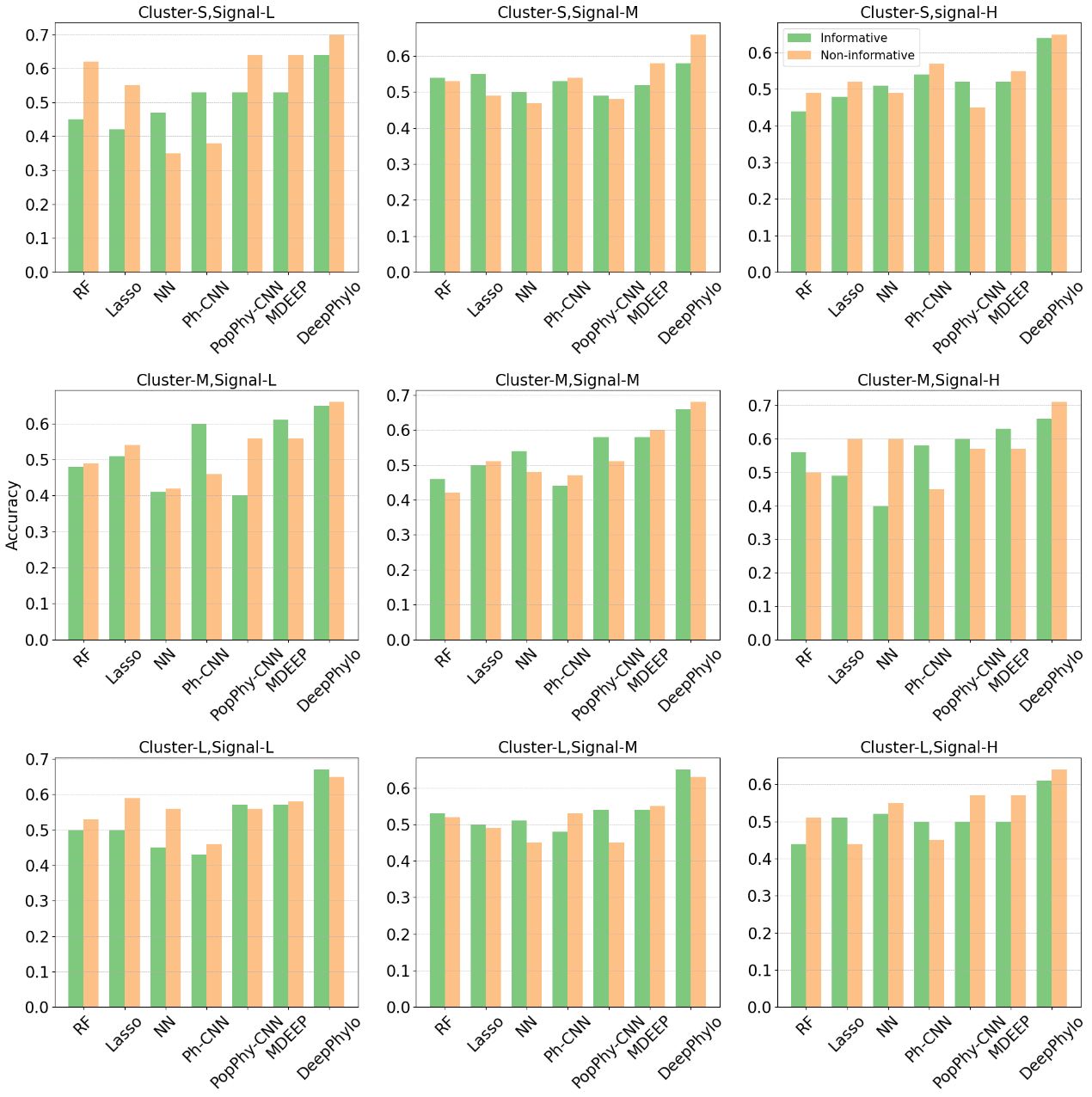


Figure S1. Model performance on simulation datasets evaluated using accuracy (ACC) as the metric, across different cluster sizes (Small, Medium, Large), signal densities (Low, Medium, High), and levels of phylogenetic tree informativeness (informative vs. non-informative).


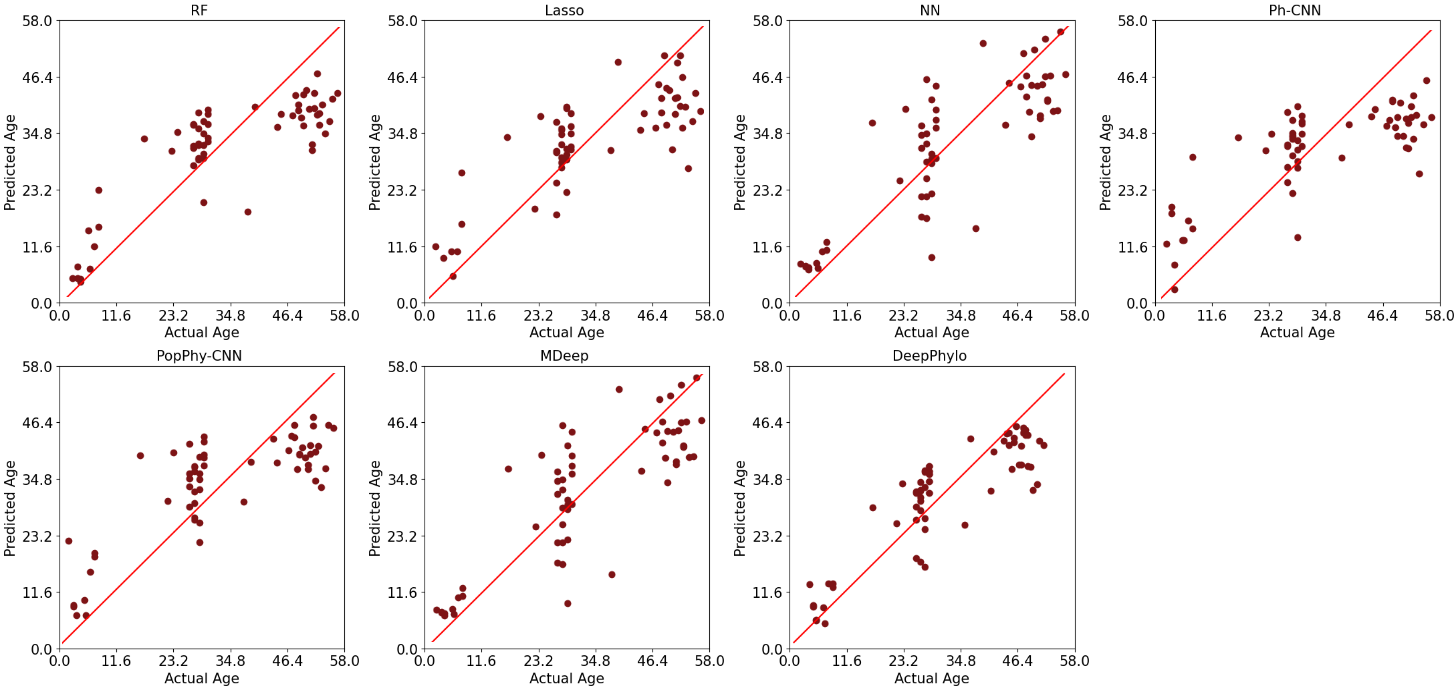


Figure S2. Detailed age regression prediction results of DeepPhylo and comparative methods.


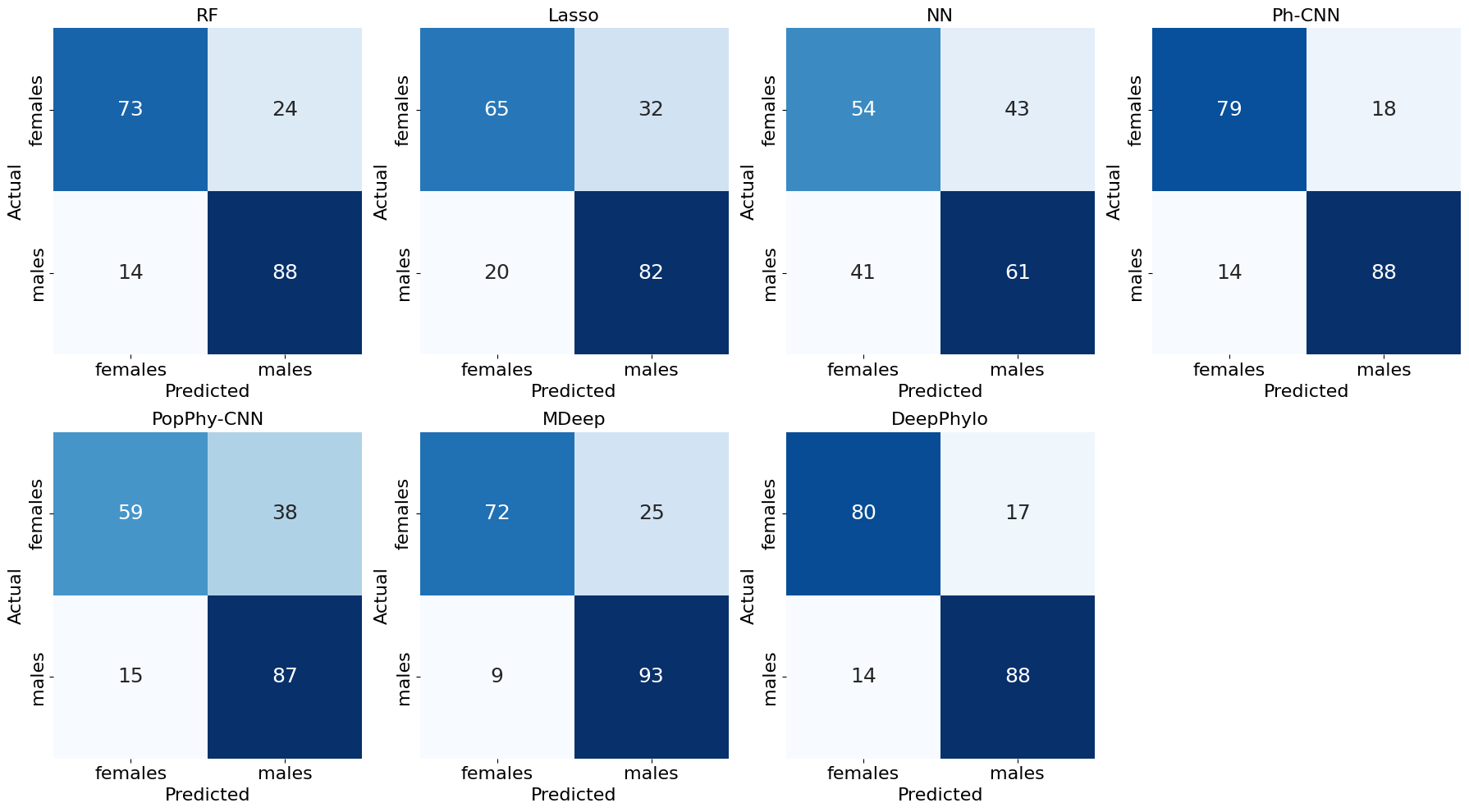


Figure S3. Detailed gender classification prediction results of DeepPhylo and comparative methods.
